# Low-Grade Papillary Schneiderian Carcinoma: A Case Report

**DOI:** 10.1007/s12105-017-0832-z

**Published:** 2017-06-21

**Authors:** Hui-Jeong Jeong, Jin Roh, Bong-Jae Lee, Kyung-Ja Cho

**Affiliations:** 1 Department of Pathology, Asan Medical Center, University of Ulsan College of Medicine, 88, Olympic-ro 43 gil, Songpa-gu, Seoul, 05505 South Korea; 2 Department of Otorhinolaryngology, Asan Medical Center, University of Ulsan College of Medicine, Seoul, South Korea

**Keywords:** Low-grade malignancy, Papillary sinonasal carcinoma, Schneiderian papilloma, Oncocytic papilloma

## Abstract

The nasal cavity and paranasal sinuses are covered with ciliated respiratory mucosa of ectodermal origin, known as Schneiderian epithelium, which can give rise to different types of sinonasal carcinomas. A 42-year-old woman with a history of nasal polypectomy 3 years previously presented with nasal obstruction and rhinorrhea. She was found to have a papillary mass involving the left nasal cavity, and the left maxillary and ethmoidal sinuses by radiologic examination. She underwent endonasal resection under the diagnosis of oncocytic papilloma. The resected specimen showed exuberant invasive growth of papillary or inverted architectures of epithelial cells. The neoplastic cells were very bland, showing a round to polygonal shape, low nuclear-to-cytoplasmic ratio, abundant oncocytic cytoplasm, uniform nuclei, indistinct nucleoli, and scarce mitosis. The overall features were identical to those of a recent report of a low-grade papillary Schneiderian carcinoma. The main differential diagnosis is Schneiderian papilloma, and awareness of this novel entity is important for its proper treatment.

## Introduction

Schneiderian epithelium covering the nasal cavity and paranasal sinuses gives rise to three kinds of benign papillomas and several kinds of carcinomas. Morphologically, inverted, exophytic, and oncocytic papillomas are classified as benign, but some have a potential, albeit low, for malignant transformation, most frequently to squamous cell carcinoma [1, 2]. The 2017 World Health Organization (WHO) histological classification of tumors of the nasal cavity and paranasal sinuses includes squamous cell carcinomas, lymphoepithelial carcinomas, sinonasal undifferentiated carcinomas, NUT carcinoma, neuroendocrine carcinoma, adenocarcinomas, and teratocarcinosarcoma with overtly malignant characteristics [3]. The low-grade papillary Schneiderian carcinoma, as recently described by Lewis et al. [4], is a novel type of carcinoma characterized by bland morphology similar to that of the Schneiderian papilloma, an invasive growth pattern, and a propensity for multiple recurrences and ultimate mortality. Here, we report an additional case of low-grade papillary Schneiderian carcinoma.

## Case Report

**Fig. 1:**
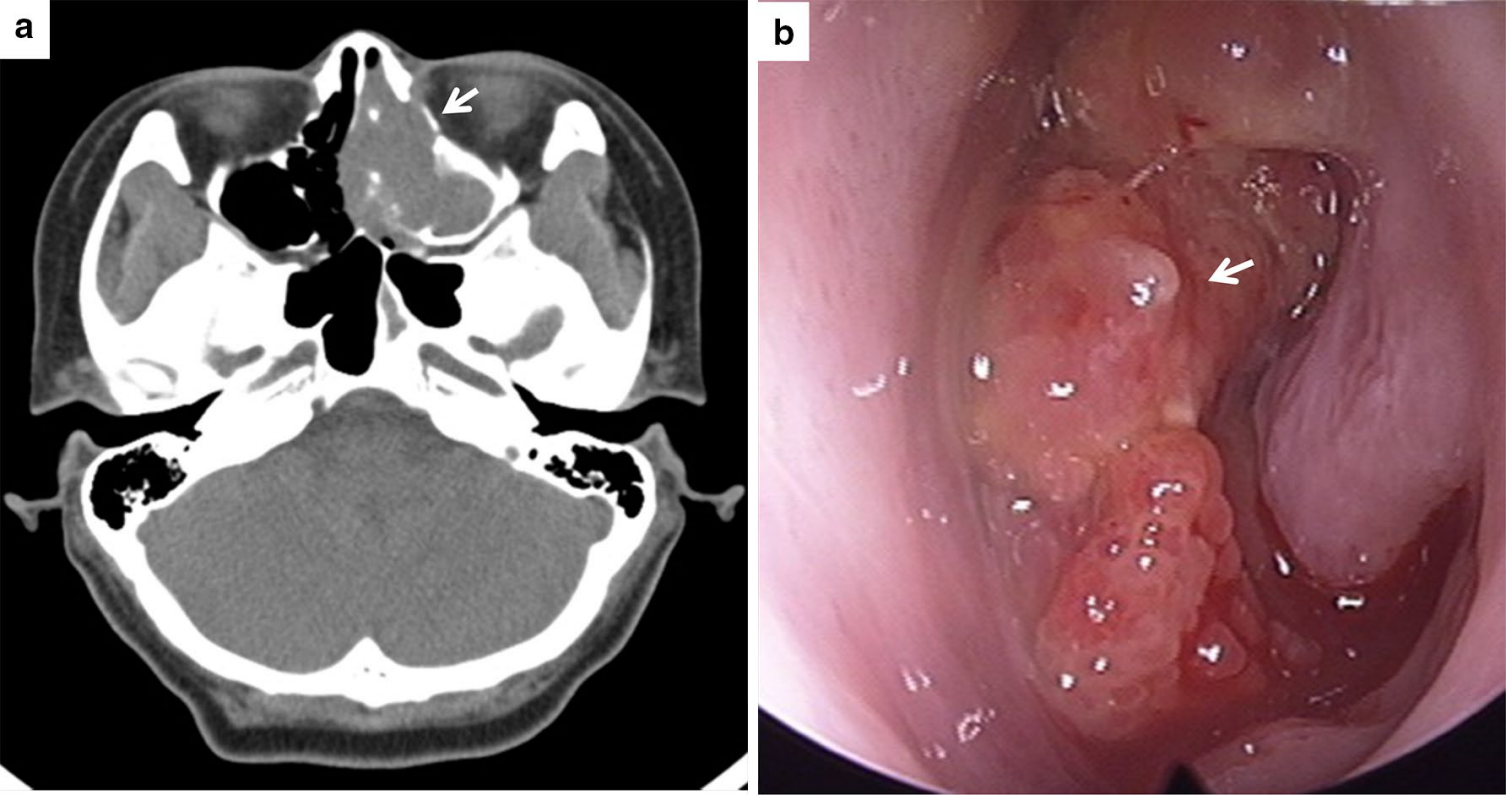
**a** Computed tomography showing a left nasal mass (*arrow*) involving the middle and inferior turbinate of the left nasal cavity as well as the medial wall of the left maxillary sinus, the lateral wall of the left maxillary sinus, and the left ethmoidal sinus and causing septal deviation. **b** A bulging mass (*arrow*) on the nasal septum was noted on nasal endoscopy

**Fig. 2:**
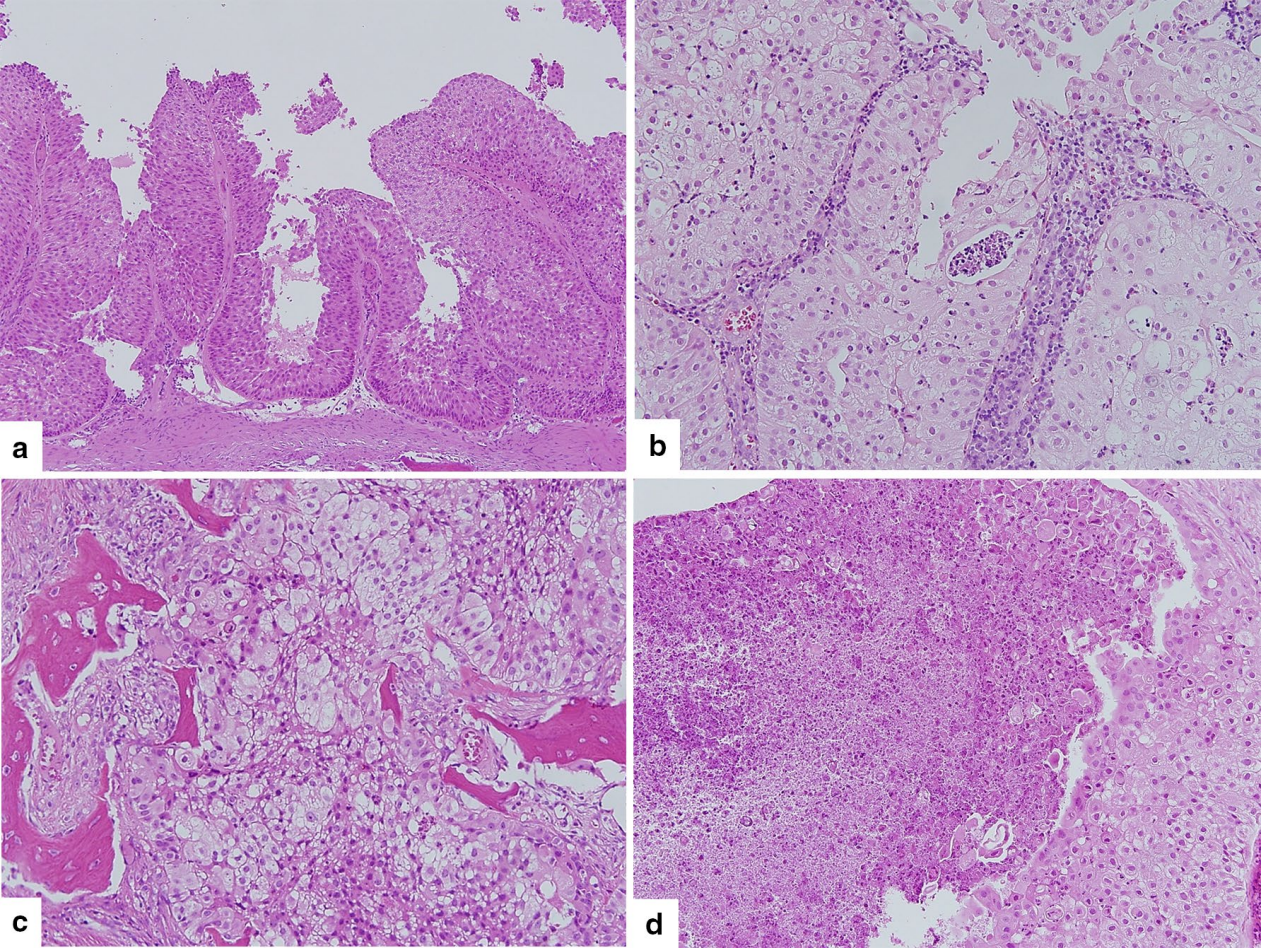
**a** Benign-looking multilayered epithelial proliferation in the biopsy specimen showing a papillary pattern. **b** Focal intraepithelial neutrophils, and microabscesses were evident. **c** Resected specimen showing diffuse bone invasion and destruction. **d** Two foci of necrosis were identified within the tumor cell nests

A 42-year-old woman presented with left nasal obstruction and rhinorrhea that had persisted for 6 months. She had already undergone polypectomy of the left nasal cavity under the diagnosis of oncocytic papilloma 3 years prior at another hospital. Based on a follow-up radiologic examination, it was apparent that the mass extent had increased over the previous 3 months. Computed tomography images revealed a heterogeneously attenuating nasal mass involving the middle and inferior turbinate of left nasal cavity, the medial and lateral walls of the left maxillary sinus, and the left ethmoidal sinus. The mass was closely abutting on the nasal septum, causing septal deviation (Fig. 1). A papillary mass with pus was noted in the left ethmoid sinus, maxillary sinus, and septum on nasal endoscopy. The bulging mass on the nasal septum led to right deviation of the septal bone and cartilage. A punch biopsy was performed and the nasal cavity mass was determined to be an oncocytic papilloma based on its papillary growth, bland morphology, slightly oncocytic cytoplasm, lacking a dense or granular appearance, round-to-oval uniform and vesicular nuclei, multilayered epithelium, focal intraepithelial microabscesses, and neutrophils (Fig. 2a, b). Subsequently, nasal endoscopic resection and septal resection with reconstruction were performed.

The resection specimen was composed of fragmented pinkish-gray soft tissue and cartilage, measured up to 5 cm in aggregates, and its cut surface was yellowish–white and solid with hemorrhage. Microscopic examination revealed extensive bone invasion of papillary or inverted architectures of epithelial cells. The neoplastic cells were very bland with a round to polygonal shape, distinct cell border, low nuclear-to-cytoplasmic ratio, abundant eosinophilic cytoplasm, uniform round-to-oval nuclei, indistinct nucleoli, and scarce mitosis (1/40 HPFs). The cytomorphologic features were reminiscent of oncocytic papilloma, but the cytoplasmic character slightly differed from that of oncocytes and the growth pattern of the neoplastic cells was invasive with evident bone destruction. The bone involvement was not pushing or erosive, but dissecting, in direct association with the epithelial proliferation (Fig. 2c). Two foci of necrosis were identified within tumor cell nests (Fig. 2d).

**Fig. 3:**
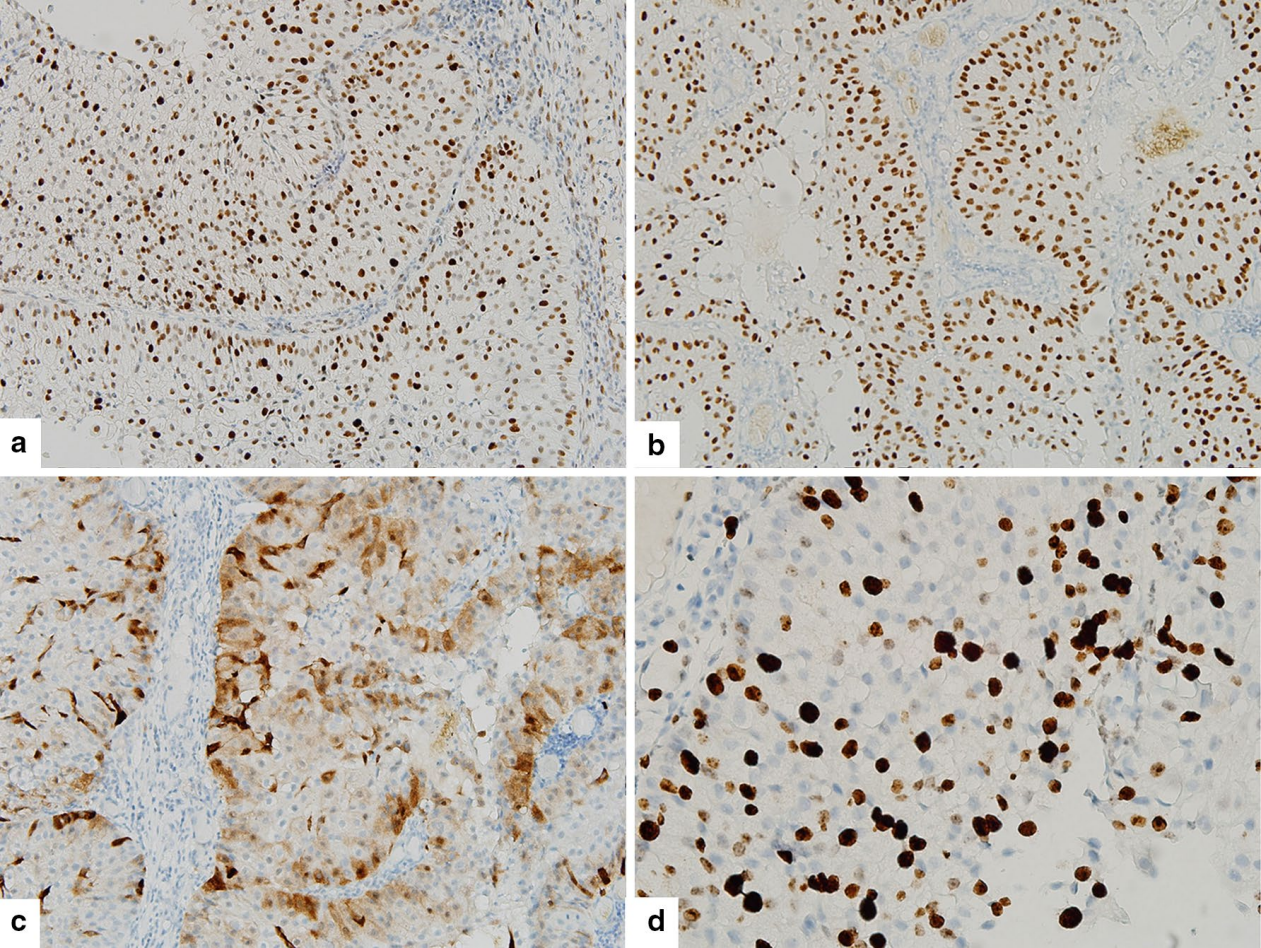
Tumor cells showed immunopositivity for p53 (50%) (**a**), and p63 (**b**), partial positivity for p16 (**c**), and a 10% Ki-67 labeling index (**d**)

Immunohistochemistry for p63 (1:200, DAKO, Glostrup, Denmark) resulted in diffuse positivity and p53 immunohistochemical staining (1:1500, DAKO) showed positivity in up to 50% of tumor cells. Immunohistochemistry for p16 (1:6, VENTANA, Tucson, AZ, USA) showed partial positivity, along the periphery of cell nests. The Ki-67 (1:200, DAKO) labeling index was low, about 10% **(**Fig. 3 **)**.

Ultimately, the patient was diagnosed with a low-grade carcinoma, and received adjuvant intensity-modulated radiation therapy because of the indeterminate resection margin. We could not review the pathology of the previous polypectomy specimen from the other hospital, but the possibility of recurrent disease could not be excluded because the lesion had been diagnosed as oncocytic papilloma. Further recurrent or metastatic disease has not been detected in a follow-up period of 21 postoperative months.

## Discussion

The Schneiderian epithelium, a special type of sinonasal epithelium, gives rise to uncommon benign tumors, known as inverted or exophytic Schneiderian (sinonasal) papillomas, which are composed of an intermediate type of epithelium between columnar and squamous epithelium, squamous epithelium, or columnar epithelium [5, 6]. Schneiderian papillomas progress to malignancy in approximately 5–10% of cases. Most carcinomas from Schneiderian papillomas are frank squamous cell carcinomas [7, 8].

Lewis et al. [4] recently described a previously undocumented pathologic case in which a 47-year-old patient who was initially diagnosed with “fungiform Schneiderian papilloma”, and died after 18 years of progressive recurrence. Over time, the mitosis level increased from <1/10 HPFs to 18/10 HPFs, and the Ki-67 labeling index increased from 5 to 50–60% in the lymph node metastasis from late in the disease course. This low-grade papillary Schneiderian carcinoma is a novel type of carcinoma characterized by a bland morphology similar to the Schneiderian papilloma, an invasive growth pattern with a pushing border, and a propensity for multiple recurrences and ultimately mortality. The authors concluded that the tumor had been a low-grade carcinoma from the beginning based on the lack of an apparent gradation from columnar to squamous epithelium.

The present case also showed invasive, but bland histomorphology, and could not be classified according to the 2017 WHO classification of tumors of the nasal cavity and paranasal sinuses including squamous cell carcinoma, lymphoepithelial carcinoma, sinonasal undifferentiated carcinoma, NUT carcinoma, neuroendocrine carcinoma, adenocarcinoma, and teratocarcinosarcoma as malignant epithelial tumors. This case was similar to the novel entity reported by Lewis et al. [4], although the clinical outcome is yet to be determined. Diffuse immunopositivity for p63, significant expression of p53, and partial positivity for p16 were also shared by the two cases. Unlike the proliferative activity, p53 positivity in Lewis’ case was described to be consistent at all time points. Although Lewis’ case showed no discernible distribution or pattern of p16 expression, our case showed positivity mainly along the periphery of tumor cell nests.

Considering the premalignant potential of Schneiderian papillomas, a possibility of carcinoma arising in a Schneiderian papilloma had to be excluded in this case. We meticulously reviewed and compared the preoperative biopsy specimen and the resection specimen, but could not discriminate between benign and malignant parts within the homogeneously bland, yet invasive, population observed throughout the tumor. Moreover, immunohistochemistry performed on both specimens showed consistent p53 positivity.

Our case has not developed recurrent or metastatic disease for 21 months after the operation at our hospital, but the present lesion might be a recurrent disease considering the patient’s history. Lewis’s case showed more than 10 recurrences over 18 years with the first recurrence occurring within 2 years. We believe that long-term close surveillance of the patient is necessary due to the aggressiveness of the tumor, and for timely treatment.

Because the current WHO classification dose not contain such an entity, the index of suspicion should generally be very low, and when such a tumor is encountered in a biopsy specimen, underdiagnosis as a Schneiderian papilloma is natural. Further reports of similar cases would increase, the awareness of a low-grade carcinoma resembling a papilloma of the sinonasal tract. A combination of careful examination of intraepithelial neutrophils and mucous cells, ancillary tests for p53, and a clinico-pathologic correlation could aid in the differential diagnosis between low-grade papillary Schneiderian carcinomas and Schneiderian papillomas [9, 10]. The mechanism of p16 expression in the present case is unknown, considering its distribution pattern, namely, partial positivity along the border of tumor cell nests. In inverted papilloma cases associated with malignancy, an adverse relationship has been found between p53 overexpression and HPV status [11, 12]. Studies of more cases are needed to determine the risk factors for this rare neoplasm.

## References

[1] Hunt JL, Bell D, Sarioglu S, El-Naggar AK, Chan JKC, Grandis JR (2017). Schneiderian papillomas. World Health Organization classification of head and neck tumours.

[2] Batsakis JG, Suarez P (2001). Schneiderian papillomas and carcinomas: a review. Adv Anat Pathol.

[3] Bishop JA, Bell D, Westra WH, El-Naggar AK, Chan JKC, Grandis JR (2017). Carcinomas of the nasal cavity, paranasal sinuses and skull base. World Health Organization classification of head and neck tumours.

[4] Lewis JS, Chernock RD, Haynes W, Ei Mofty SK (2015). Low-grade papillary Schneiderian carcinoma, a unique and deceptively bland malignant neoplasm; report of a case. Am J Surg Pathol.

[5] Kaufman MR, Brandwein MS, Lawson W (2002). Sinonasal papillomas: clinicopathologic review of 40 patients with inverted and oncocytic Schneiderian papillomas. Laryngoscope.

[6] Vorasubin N, Vira D, Suh JD, Bhuta S, Wang MB (2013). Schneiderian papillomas: comparative review of exophytic, oncocytic, and inverted types. Am J Rhinol Allergy.

[7] Nudell J, Chiosea S, Thompson LDR (2014). Carcinoma Ex-Schneiderian papilloma (malignant transformation): a clinicopathologic and immunophenotypic study of 20 cases combined with a comprehensive review of the literature. Head Neck Pathol.

[8] Anari S, Carrie S (2010). Sinonasal inverted papilloma: narrative review. J Laryngol Otol.

[9] Mumbuc S, Karakok M, Baglam T, Karatas E, Durucu C, Kibar Y (2007). Immunohistochemical analysis of PCNA, Ki67 and p53 in nasal polyposis and sinonasal inverted papillomas. J Int Med Res.

[10] Fan GK, Imanaka M, Yang B, Takenaka H (2006). Characteristics of nasal inverted papilloma and its malignant transformation: a study of cell proliferation and programmed cell death. Am J Rhinol.

[11] Franzmanna BM, Buchwaldb C, Jacobsena GK, Lindebergc H (1998). Expression of p53 in normal nasal mucosa and in sinonasal papillomas with and without associated carcinoma and the relation to human papillomavirus (HPV). Cancer letter.

[12] Buchwald C, Lindeberg H, Pedersen BL, Franzmann MB (2001). Human papilloma virus and p53 expressionin carcinomas associated with sinonasal papillomas: a Danish epidemiological study 1980–1998. Laryngoscope.

